# PET-guided early identification of CAR-T candidates in diffuse large b-cell lymphoma: a multidisciplinary expert consensus

**DOI:** 10.1007/s00259-026-07999-9

**Published:** 2026-06-26

**Authors:** Luca Guerra, Domenico Albano, Piera Angelillo, Alice Di Rocco, Mirko Farina, Andrea Farolfi, Vittoria Tarantino, Carlo Visco, Pier Luigi Zinzani

**Affiliations:** 1 School of Medicine and Surgery, University of Milano Bicocca, Milan, Italy; 2 Nuclear Medicine Unit, IRCCS Fondazione San Gerardo dei Tintori, Monza, Italy; 3 Department of Nuclear Medicine, University of Brescia, Brescia, Italy; 4 Department of Nuclear Medicine, ASST Spedali Civili, Brescia, Italy; 5 Hematology and BMT Unit, Lymphoma Unit, IRCCS San Raffaele Scientific Institute, Milano, Italy; 6 Hematology, Department of Traslational and Precision Medicine, University SAPIENZA of Rome, Rome, Italy; 7 AOU, UOC Ematologia e Trapianto, Policlinico Umberto 1, Rome, Italy; 8 Unit of Blood Diseases and Bone Marrow Transplantation, Cell Therapies and Hematology Research Program, Department of Clinical and Experimental Science, ASST- Spedali Civili di Brescia, University of Brescia, Brescia, Italy; 9 Nuclear Medicine, IRCCS Azienda Ospedaliero-Universitaria di Bologna, Bologna, Italy; 10 Division of Hematology, Azienda Ospedaliera Ospedali Riuniti Villa Sofia- Cervello, Palermo, Italy; 11 Hematology and Bone Marrow Transplant Unit, Section of Biomedicine of Innovation, Department of Engineering for Innovative Medicine, University of Verona, Policlinico G.B. Rossi, Verona, Italy; 12 Department of Medicine and Surgery, Bologna University, Bologna, Italy; 13 IRCCS Azienda Ospedaliero-Universitaria di Bologna, Istituto di Ematologia “Seràgnoli”, Bologna, Italy

**Keywords:** DLBCL, lymphoma, PET/CT, FDG, CAR T-cells

## Abstract

**Purpose:**

This expert consensus aims to define how 2-deoxy-2-[18 F]-fluoro-D-glucose ([¹⁸F]FDG) positron emission tomography/computed tomography (PET/CT) can be systematically integrated into clinical pathways for early identification of diffuse large B-cell lymphoma (DLBCL) patients who may benefit from chimeric antigen receptor T-cell (CAR-T) therapy. The main research question is how PET-derived parameters and timing can optimize risk stratification and guide timely referral for advanced therapies.

**Methods:**

A multidisciplinary panel of nuclear medicine physicians and hematologists conducted a structured consensus process based on current literature, international guidelines, and expert discussion. Key clinical scenarios and statements were evaluated across multiple meetings to assess the role of PET/CT at baseline, interim, and end-of-treatment stages.

**Results:**

PET/CT provides critical prognostic information throughout the disease course. Baseline quantitative metrics (e.g., metabolic tumor volume) improve risk stratification beyond conventional indices. Interim PET, particularly using Deauville score and the change in the maximum standardized uptake value, enables early identification of high-risk or refractory patients. End-of-treatment PET remains essential for response assessment and therapeutic decision-making. Standardization of acquisition, reporting, and interpretation is necessary to enhance reproducibility and clinical integration.

**Conclusion:**

PET/CT should be considered a central biomarker in DLBCL management, enabling earlier identification of high-risk patients and facilitating timely CAR-T referral. Harmonized, multidisciplinary implementation is essential to optimize outcomes and improve access to potentially curative therapies.

## Introduction

Diffuse large B-cell lymphoma (DLBCL) is the most common subtype of non-Hodgkin lymphoma (NHL) and remains associated with a substantial proportion of patients who are refractory to first-line therapy (primary refractory) or experience early relapse (within 6 months after a complete response) [1, 2]. Approximately 30–40% of DLBCL patients develop relapsed or refractory (R/R) disease after first-line rituximab-based chemoimmunotherapy. More specifically, approximately 10–15% have primary refractory disease (incomplete response or relapse within 6 months), and an additional 20–25% relapse after an initial response, typically within the first 2 years [3–5]. This translates into a substantial proportion of patients requiring second-line and subsequent therapies. Outcomes in R/R LBCL without modern therapies are historically dismal. The landmark SCHOLAR-1 study of 636 patients with refractory DLBCL demonstrated an objective response rate of only 26% (complete response [CR] 7%) to the next line of therapy, with a median overall survival (mOS) of just 6.3 months and only 20% alive at 2 years [6]. In this clinical scenario, chimeric antigen receptor T-cell (CAR-T) therapy has emerged as a transformative treatment option for relapsed or refractory disease, dramatically increasing the rate of response and the prognosis in a subset of patients [7].

A recent meta-analysis of second-line trials (ZUMA-7, TRANSFORM, BELINDA) and including 1,199 patients, confirmed that CAR T-cell therapy significantly improves OS (hazard ratio [HR] 0.75), event free survival (EFS) (HR 0.51), and progression free survival (PFS) (HR 0.47) compared to standard of care, with 3-year OS of 53.6% vs. 41.5% [8]. In details, in the ZUMA-7 trial median (m) OS was nor reached (NR) and mPFS was 14.4 months, in the TRNSFORM trial mOS and mPFS were NR and mEFS was 29.5, in the Belinda trial mEFS was 3.0 months, respectively [9–11]. Moreover, real-world data support a curative potential in approximately 40–50% of patients treated with CAR T-cell therapy [12].

However, the clinical benefit of CAR-T therapy is strongly influenced by timely patient identification and referral, which must be anticipated early in the disease course and often before the exhaustion of conventional therapeutic options [13–15]. Consequently, the evaluation of the risk of R/R disease in naïve DLBCL patients is critically important in the CAR-T era, as it directly informs treatment sequencing, to early identifies patients who are most likely to experience primary refractory disease or early relapse, the actual patients who benefit most from CAR T-cell therapy for timely planning this treatment [16].

2-deoxy-2-[18 F]-fluoro-D-glucose ([¹⁸F]FDG) positron emission tomography/computed tomography (PET/CT) plays a central role in the management of DLBCL and is routinely used at diagnosis, during treatment, at the end of therapy (EOT) and at follow-up when applicable [17]. Beyond its established role in response assessment, [¹⁸F]FDG PET/CT has become increasingly relevant for CAR-T-oriented decision-making, supporting the evaluation of disease burden, metabolic activity, and anatomical distribution before infusion, as well as early response and prognostication after therapy [18]. In this context, nuclear medicine physicians are not only involved in treatment response evaluation but also act as key contributors to multidisciplinary therapeutic planning, working in close integration with hematologists [18].

Growing evidence demonstrates that baseline PET-derived semiquantitative parameters - such as maximum standardized uptake value (SUVmax), metabolic tumor volume (MTV), and total lesion glycolysis (TLG) - provide robust prognostic information in DLBCL patient disease presentation [19–21]. In addition to baseline PET/CT, interim PET/CT (typically after 2–4 cycles of therapy) provides prognostic information, especially when quantitative measures such as the change in maximum standardized uptake value (ΔSUVmax) are used; a poor metabolic response is associated with inferior PFS and OS [22–24]. Finally, EOT PET/CT, interpreted using the Deauville five-point scale, is standard for response assessment, with a robust prognostic value: commonly a Deauville score (DS) of 1–3 indicates complete metabolic response (CMR) and is associated with favorable long-term prognosis, while patients with scores of 4–5 had a higher prognostic risk [3, 25].

PET/CT plays a role also in patients who experienced relapse or refractoriness and are candidate to CAR-T treatment. High baseline MTV, TLG, and SUVmax have consistently been associated with inferior PFS and OS, lower response rates, and increased risk of treatment-related toxicities, including cytokine release syndrome [26–28]. Furthermore, early post-infusion PET/CT, particularly at 1 month, allows refined risk stratification through assessment of metabolic response dynamics, including ΔSUVmax (percentage reduction in maximum standardized uptake value from baseline) and changes in tumor burden, enabling early identification of patients at risk of treatment failure or relapse [27].

Despite the increasing body of evidence supporting the prognostic and predictive value of PET/CT in DLBCL, there is currently no shared expert consensus specifically addressing how PET findings should be systematically integrated into CAR-T referral pathways and therapeutic decision-making. Existing guidelines primarily focus on response assessment and do not provide clear recommendations on the optimal timing of PET examinations, the essential quantitative parameters to be reported, or their interpretation in the context of early CAR-T planning. As a result, imaging practices, reporting standards, and clinical integration of PET-derived information remain heterogeneous across Centers [29].

Additional challenges further complicate PET interpretation in this setting. False-positive findings related to inflammation, infection, or immune-mediated activation are frequent also after CAR-T therapy, while pseudo progression, although uncommon, represents a potential diagnostic pitfall [30]. Moreover, PET/CT does not directly reflect CAR T-cell expansion or persistence, and the use of bridging therapies (BT) prior to infusion may be a confounding variable in the metabolic assessment [31, 32]. Prospective evidence on optimal PET timing after bridging therapy (BT), when performed, is limited and BT strategies are heterogeneous; therefore, no standardized interval is established. PET/CT should be performed after BT completion and before lymphodepletion, as close as feasible to CAR-T infusion, to reflect current disease burden and to exclude central nervous system involvement and/or critical progression of disease. Imaging should be scheduled when the patient is clinically stable, avoiding very early scans. Interpretation must consider BT type and supportive treatments, such as growth factors, which may influence tracer uptake.

These limitations highlight the need for standardized, consensus-driven interpretative frameworks. Although the body of evidence, several clinically relevant questions remain unresolved. For example, the prognostic significance of a partial metabolic response (PR) at interim PET followed by a CMR at EOT imaging is still debated [33]. Similarly, the extent to which longitudinal changes in PET-derived quantitative parameters can reliably identify patients at increased risk of early relapse or refractoriness, and how this information should guide follow-up strategies and early CAR-T pathway activation, has yet to be clearly defined.

The present expert consensus aims to address these unmet needs by providing a structured, multidisciplinary, and imaging-centered framework for the use of PET/CT in DLBCL patients with potential eligibility for CAR-T therapy. Through a critical review of the literature and expert panel discussion, this consensus seeks to define optimal PET timing, essential PET-derived parameters to be included in clinical reports, deal with methodological issues to improve reproducibility of PET/CT results and evaluate the implications of all these factors for CAR-T-oriented therapeutic planning [29]. In fact, only about one third of eligible patients received CAR-T, demonstrating the importance of early and effective patient identification and referral [34]. By emphasizing the interaction between nuclear medicine physicians and hematologists, this document aims to support harmonized clinical practice across both referral and hub Centers, promoting earlier identification of high-risk patients and more timely access to CAR-T therapy.

## Methods

The Scientific Committee, based on existing literature, current guidelines [35, 36], and preliminary Experts’ inputs, identified the key factors necessary to evaluate the appropriateness of each intervention and discussed a set of clinical scenarios/statements which were evaluated for their risk/benefit profile through three separate meetings till the reaching of the consensus of the recommendations that follow in the subsequent sections. The Expert Panel was formed with 9 members including 2 project leaders (one nuclear medicine physician and one hematologist). The Panel consisted of both experienced nuclear medicine physicians and hematologists from different Italian geographical representations, as showed by their publications, role in clinical trials relevant to the present issues, and involvement in national and international associations with also task of guidelines writing. Their expertise covered a broad spectrum of clinical and research perspectives, reflecting real-world clinical practice in various regions of Italy, encompassing the northern, central, and southern areas of the Country. Clinical expertise from both referral and tertiary Centers was incorporated in the consensus process.

### Baseline [¹⁸F]FDG PET/CT

Baseline [¹⁸F]FDG PET/CT is the standard imaging modality for initial staging and risk assessment in DLBCL. [¹⁸F]FDG PET/CT offers superior sensitivity and specificity compared to conventional CT, particularly for detecting extranodal involvement, which is present in up to two-thirds of DLBCL cases [3, 37]. This accuracy enables precise Ann Arbor staging and mapping of disease sites, which is essential for subsequent response assessment and treatment planning [3, 38, 39].

Baseline [¹⁸F]FDG PET/CT eliminates the need for routine bone marrow (BM) biopsy in selected patients, as it reliably detects marrow involvement, except in cases of low-volume or discordant indolent disease [3, 37]. This streamlines the diagnostic process and reduces patient burden.

Quantitative PET metrics at baseline, including MTV, TLG, and lesion dissemination (like Dmax), provide robust prognostic information. High MTV and TLG are independently associated with inferior PFS and OS, regardless of clinical risk factors such as age or performance status [22, 24, 40–43]. Dmax, reflecting the spatial spread of disease, complements MTV and further refines risk stratification; patients with both high MTV and high Dmax have markedly worse outcomes [40, 42, 44–46]. While quantitative PET metrics such as MTV, TLG, and Dmax provide significant prognostic value, they currently lack consolidated and universally accepted cut-off values for routine clinical use, even more in a prospective potential CAR-T cell approach. 

These PET-derived features outperform traditional prognostic indices such as the international prognostic index (IPI) in predicting relapse and survival [42, 45]. Recent models combining baseline PET features with clinical data (e.g., age, performance status) and interim PET response have demonstrated superior predictive accuracy for treatment failure and survival. Dynamic risk tools incorporating MTV, Dmax, and early PET response (ΔSUVmax) enable identification of very high-risk patients who may benefit from alternative or intensified therapies [45]. Although standardization of segmentation method has been recently defined [47], the lack of consolidated cut-off values and of widespread and authorized clinical practice software for PET metrics calculation may represent a limitation to the implementation of MTV in clinical practice [24, 43, 48]. The most common segmentation methods are based on fixed absolute SUV thresholds (e.g., SUV ≥ 2.5 or SUV ≥ 4.0) or on a percentage of the SUVmax (e.g., 41% of SUVmax) [49]. Several studies reported comparable prognostic performance of MTV regardless of the segmentation method applied, suggesting that the choice of approach may not be a critical determinant [50]. The adoption of an SUV threshold of 4.0 has been supported by its high reproducibility and its practicality for routine clinical implementation [47].

### Baseline Staging before first-line

[18 F]FDG PET/CT is strongly recommended for the initial staging of DLBCL (Class I, Level of Evidence A) as it provides comprehensive evaluation of nodal and extranodal disease, detection of BM involvement, identification of metabolically active lesions not evident on CT scan, Accurate Ann Arbor staging, direct impact on treatment planning [35, 51]. Compared with contrast-enhanced CT alone, PET/CT demonstrates superior sensitivity, particularly in bone and BM involvement, splenic disease and extranodal sites with minimal structural changes [51].

Additional PET-derived parameters, such as bone marrow retention index (RI-bm) and bone marrow-to-liver ratio (BLR), have emerged as independent predictors of relapse-free and overall survival, offering noninvasive risk assessment in newly diagnosed DLBCL [52].

### Baseline staging and assessment in subsequent lines

Because of its improved sensitivity, [18 F]FDG PET/CT findings may lead to a reassessment of disease stage, which in turn can influence clinical management.

If areas of increased [¹⁸F]FDG uptake are detected but do not correspond to the typical clinical pattern of lymphoma, additional diagnostic procedures, including further clinical assessment or histopathological confirmation, are recommended [51, 53].

Baseline PET/CT is essential for accurate staging, risk stratification, and prognostication in DLBCL. Semiquantitative PET metrics (MTV, TLG, Dmax, RI-bm, BLR) should be integrated into clinical risk models to optimize patient management, although further harmonization and prospective validation are needed for routine clinical use [51].

### Baseline PET/CT: reporting metrics and timing

Baseline MTV should be reported on routine staging PET/CT in DLBCL; the use of this quantitative parameter is strongly supported by prognostic evidence. Baseline MTV is consistently an independent prognostic factor for PFS and OS in DLBCL, often outperforming or adding to the IPI. The International Metabolic Prognostic Index (IMPI), combining MTV with age and stage, outperformed IPI in defining high-risk groups (3-year PFS 46.3% vs. 58.0%; 3-year OS 51.5% vs. 66.4%) [54]. MTV also provides additive prognostic value within NCCN-IPI risk groups, particularly further stratifying high-risk patients (NCCN-IPI ≥ 4) into subgroups with significantly different outcomes [55, 56]. In the relapsed/refractory setting, including pre-CAR T-cell therapy, MTV outperforms IPI for predicting PFS [57]. A recent review recommends that risk-adapted, biologically driven care should incorporate imaging disease burden where feasible [58].

Reporting MTV on baseline staging PET/CT in DLBCL is increasingly feasible and clinically informative. Using a fixed SUV threshold of 4.0 with semiautomated segmentation software provides the most reproducible and practical approach for routine clinical use [37, 47]. While MTV does not yet formally alter first-line treatment selection in guidelines, it can inform prognostic discussions, clinical trial eligibility, and when defined before CAR-T infusion may guide decisions regarding CNS prophylaxis [40].

**Fig. 1:**
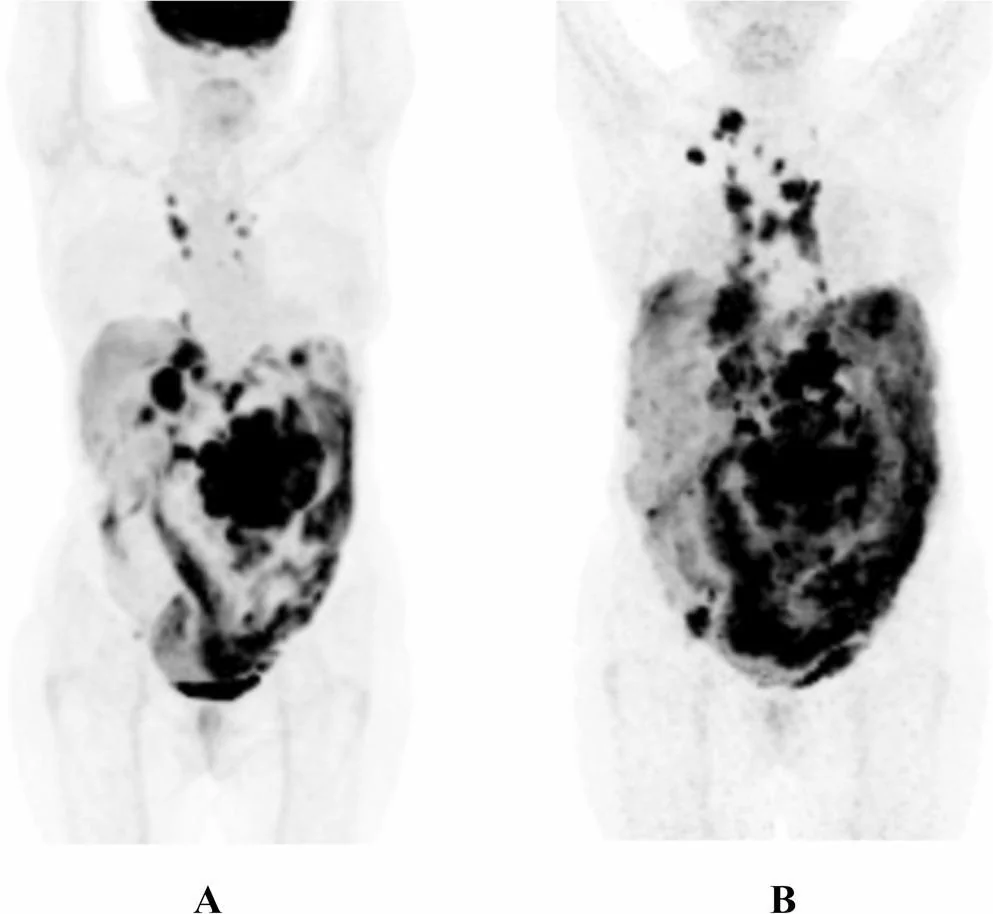
A 77yrs old woman with a stage III diffuse large B-cell lymphoma according to PET executed in 22/12/2025 (**A**). Due to a rapid deterioration of clinical status (fever, weight loss, increased lactate dehydrogenase, asthenia) the patient was re-evaluated with PET one month after, immediately before starting the therapy (**B**) that demonstrated nodal disease progression, diffuse peritoneal spread, pleural effusion and probable bowel involvement with upstaging to stage IV. The TMTV (total metabolic tumor volume) value rose from 698 ml (**A**) to 1040 ml (**B**)

A consideration regarding the time interval between baseline PET imaging and initiation of first-line therapy in DLBCL is due. Although no specific guidelines define a maximum acceptable interval between staging PET and treatment initiation [30, 59], it should be considered that the proliferative rate (Ki-67%) in DLBCL could be very high (70% to > 90%). This biological feature may contribute to a rapid disease progression over a matter of weeks, especially when associated with worsening clinical status (Fig. 1). Consequently, if the interval time between baseline PET/CT and therapy initiation is too long, the evaluation of the true response to treatment could be incorrectly evaluated.

### Interim PET/CT response evaluation

Beyond its role in initial staging, [¹⁸F]FDG PET/CT is also widely applied for evaluating response to treatment. The technique demonstrates high sensitivity and specificity in identifying residual disease. According to the National Comprehensive Cancer Network (NCCN) Guidelines and EHA Clinical Practice Guidelines, interim evaluation with [¹⁸F]FDG PET/CT is recommended in the management of patients with DLBCL, providing important diagnostic and prognostic information, particularly for predicting PFS and OS. For interpretation and clinical reporting of interim PET findings, the Deauville 5-point scale is highly recommended [30, 58]. This scoring system evaluates the intensity of [¹⁸F]FDG uptake in lymphoma lesions relative to reference uptake in the mediastinal blood pool and liver and it is currently considered a standard of care for the clinical management of the patients as the DS (and the Lugano classification) represent the common language through which the nuclear medicine physician communicates to the clinician the patient’s response to therapy, and upon which the clinician bases the treatment strategy.

In addition to the DS, the ΔSUVmax, defined as the percentage difference between the SUVmax of the most avid lesion at baseline and that of the most avid residual lesion in interim PET (not necessarily the same lesion), represents a semiquantitative parameter that can further refine patient stratification, particularly among those who do not achieve CMR according to the DS (i.e. DS 1–3). A ΔSUVmax% ≥ 66% after 2 cycles and ≥ 70% after four cycles of chemo-immunotherapy, is associated with an adequate treatment response and a more favorable prognosis and reduced false-positive interpretations [60, 61]. However, these values are not yet fully validated as actionable clinical cutoffs. Thus, treatment escalation or referral for CAR-T therapy based solely on these quantitative reductions without histological confirmation is not recommended.

When PET/CT results are used to guide therapeutic decisions, particularly in patients who do not reach CMR, a biopsy is advised in order to confirm as true positive the PET/CT findings before changing the treatment strategy [62]. Additionally, in patients with CMR in interim PET and undergoing a predefined treatment protocol, the initially planned therapy should generally be completed.

Experts recommended interim PET/CT after 3–4 cycles of first-line therapy in all patients, regardless of risk profile. In cases of suspected disease progression before completion of four cycles, PET imaging may also be performed earlier [62].

As above mentioned, for response evaluation criteria, the Lugano classification is mandatory [37, 63]; the DS 1–3 is commonly considered a CMR, whereas 4–5, with reduced uptake compared to previous scan, a PMR; DS 5 with increased uptake compared to previous scan or new FDG-avid lesions consistent with lymphoma is classified as progressive metabolic disease (PMD). This modality of response assessment by the nuclear medicine physician is mandatory as it allows clinicians to identify patients not CMR, who can be considered chemorefractory (i.e. when only chemotherapy is used) [14] and timely evaluated for CAR-T therapy or referred to a hub CAR-T Center.

Importantly, there are significant prognostic differences between DS 4 and DS 5 on interim PET/CT in DLBCL, with DS 5 carrying a substantially worse prognosis than DS 4. A recent comprehensive systematic review and meta-analysis (44 studies) specifically addressed this question and demonstrated that the pooled PFS HR for DS 5 alone was 5.59 (95% confidence interval [CI], 4.35–7.19) compared to 2.36 (95% CI, 1.74–3.21) for the combined DS 4–5 positive group. For OS, the difference was even more striking: HR 8.10 (95% CI, 6.04–10.85) for DS 5 alone versus 3.31 (95% CI, 2.09–5.24) for DS 4–5 combined [64]. This discrepancy between DS 4 and DS 5 in terms of prognosis may be due to the higher FP rate in DS 4, as reported in UK NCRI and PETAL trials [65, 66]. In this case, the previously mentioned ΔSUVmax% evaluation can be more appropriate to assess if the response is adequate or not; it is interesting to note that the 66% thresholds after 2 cycles is equivalent to a qPET value (the SUV ratio among reference lesion and liver) very similar to DS 5 on the visual scale [66]. Another interesting point of interim DS 5 is the conversion rate from interim DS5-PMR to a DS1/3-CMR at EOT; currently, the data are limited but the best direct evidence comes from the UK NCRI prospective study by Mikhaeel et al., which provides DS-stratified EOT CR rates: only 36.4% of patients with interim DS 5 achieved CR at EOT [63]. Moreover, Gao et al. demonstrated a critical distinction among PMR patients (DS 4–5 combined): overall, approximately 62.5% converted to CMR at EOT [33]; however, when isolating DS 5 specifically, the conversion rate drops substantially to approximately one-third, reflecting the much worse prognostic significance of DS 5 compared to DS 4. In conclusion, DS 5 on interim PET should be considered the more reliable threshold for identifying truly high-risk patients who may benefit from second-line treatment, specifically CAR-T, whereas DS 4 may frequently represent post-treatment inflammation rather than active disease and treatment escalation based solely on DS 4 may lead to unnecessary intensification. Moreover, combining the DS with ΔSUVmax (e.g. DS 4–5 plus ΔSUVmax ≤ 70%) can further refine risk stratification and identify an extremely high-risk subgroup (about 10% of patients) with very poor prognosis eligible for second-line treatment, as CAR T-cells [22]. From a clinical practice point of view, the above-mentioned points rise several important considerations about the elements that should be included in an interim PET report, as they may be potentially useful for the hematologist: the DS value, the SUVmax of the most active lesion (reference lesion) and its anatomical location both in interim and baseline PET/CT, the ΔSUVmax, and the SUVmax of the liver (i.e. the threshold separating DS 1–3 from DS 4–5) are key elements for the clinicians and they should always be reported in the PET/CT clinical report.

### Interim PET/CT: reporting metrics and timing

To optimize the timing of interim PET execution and considering that - barring unforeseen events or complications - therapy follows well-defined time schedules, it is advisable to schedule the interim reassessment at the start of treatment. Specifically, if interim PET/CT is programmed after the fourth treatment cycle, it should be scheduled timely as close as possible to the start of the subsequent cycle (fifth), in order to provide the most representative assessment of the disease response.

Regarding the PET/CT metrics to reported in clinical report, the DS value of the reference lesion (the more FDG avid residual lesion), its anatomical localization and SUVmax, the highest SUVmax in baseline PET/CT (to derive the ΔSUVmax), are fundamental elements to be reported in a clinical interim PET/CT report; based on these elements, the hematologist is able to stratify the risk of relapsed or refractory disease and, if necessary, identify patients who may require closer monitoring in view of second-line treatment, such as CAR T-cell therapy. Liver SUVmax should also be integrated in PET/CT report as a semiquantitative parameter, helping to ensure that observed SUV changes in tumors reflect true biologic change rather than technical drift.

### End-of-treatment response evaluation

EOT PET/CT is strongly recommended in DLBCL (Class I, Level A) [49]. Its clinical utility lies in the differentiation between residual fibrosis and viable tumor, confirmation of CR and early identification of progressive disease (PD). Interpretation follows the Lugano classification, incorporating metabolic criteria based on the DS [63, 67]. The optimal timing in case of high-risk patients scheduled for CAR-T treatment is after the end of the first line (ideally within 1 month after the administration of the last cycle or at the last cycle of chemoimmunotherapy, the 6th for patients who complete the schedule), but it is needed to take into account that if interim PET is performed after the fourth cycle, some patients refuse to repeat imaging so closely. Since some patients might refuse a PET scan after only 2 cycles, in these cases the right timing for an interim assessment could be performed earlier (at 2nd or 3rd cycles).

EOT PET/CT is a central modality for response assessment in DLBCL following first-line immunochemotherapy. EOT PET CMR is a strong independent predictor of PFS and OS, regardless of baseline risk factors or cell of origin [33]. In the GOYA phase 3 trial, patients achieving PET-based CMR at EOT had significantly improved PFS and OS compared to those with residual metabolic activity, with hazard ratios of 0.26 for PFS and 0.12 for OS, confirming the prognostic value of EOT PET across a large cohort [20].

As in interim PET/CT, visual assessment of response is based on 5 points DS, but quantitative metrics such as the lesion-to-liver SUV ratio (LLR) have demonstrated superior specificity and accuracy for predicting disease progression and death [68]. An LLR cutoff of 1.83 has been validated to distinguish high-risk patients, with positive EOT PET associated with significantly worse outcomes [69]. Multivariate analyses confirm that EOT PET positivity by these criteria is independently associated with unfavorable PFS and OS. Notably, the LLR threshold of about 1.8 is very similar to the definition of DS 5 as stated by the Lugano classification and the Prolog Consensus Initiative that indicates a DS 5 when SUVmax of the residual lesion is > 2 x SUVmax liver [63, 70]. This is confirmed also by Li et al. who showed that DS 5 PFS and OS curves were very similar to those obtained when LLR > 1.83 was applied [14]. Accordingly, consistent with the considerations outlined for interim PET, a DS of 5 at EOT PET appears to represent the most reliable parameter for identifying patients with residual disease and an unfavorable prognosis. In any case, if treatment escalation is being considered, a biopsy of the residual lesion is necessary to confirm the presence of disease.

EOT PET/CT also guides post-treatment management, including the selective use of consolidative radiotherapy (RT) in patients with residual PET-positive masses. In advanced-stage DLBCL, PET-guided RT for EOT PET-positive patients yields outcomes approaching those of PET-negative patients, while PET-negative patients with bulky disease can safely omit RT without compromising survival.

At the end of first-line therapy, patients should be classified as chemorefractory in the presence of a DS of 4 or 5 on FDG-PET. In this context, CAR T-cell therapy should be considered for patients with PD, stable disease (SD), or PR at the completion of first-line treatment, and eligibility should be assessed as early as possible [14]. If this patient is undergoing RT, such an approach could be considered as a BT to CAR-T.

In summary, EOT PET is essential for prognostication and post-treatment decision-making in DLBCL, with both the DS and the Lugano criteria validated in large clinical trials and currently representing the reference standard for the interpretation and reporting of PET/CT images for treatment response assessment. Regarding the PET/CT metric in the clinical report, SUVmax of the reference lesion and highest SUVmax in the baseline scan are always recommended. EOT PET-negative patients (DS 1–3) have excellent outcomes and may avoid unnecessary additional therapy, while EOT PET-positive (DS 4–5) patients require individualized management strategies, and usually a biopsy conformation of the residual disease and when positive a second line treatment, including CAR-T approach.

## Methodological consideration

When quantitative parameters are used in PET/CT, particularly SUVmax, it is necessary to consider factors that can improve the reproducibility of the measurement.

To maximize SUVmax reproducibility across longitudinal PET/CT scans in DLBCL patients, several critical methodological domains must be rigorously controlled. The available evidence identifies some key factors, but we focus mainly to those related to scanner and acquisition protocol and images reconstruction:

Consistent Uptake Time (Injection-to-Scan Interval): SUV in metabolically active tumors continues to rise well beyond 60 min post-injection; usually an uptake period of at least 60+/- 5 min is the standard, but whatever interval is chosen must be replicated quite precisely [48, 71].

### Scanner, acquisition protocols and reconstruction parameters

Scanners and reconstruction parameters can significantly affect SUV measurements, then serial scans should be performed on the same PET/CT scanner (ideally at the same center) using identical acquisition mode, reconstruction algorithms, filters, matrix size, and attenuation correction methods. To improve reproducibility among different nuclear medicine centers, the NCCN Hodgkin Lymphoma guidelines (applicable principles extended to LBCL) recommend that PET/CT be acquired in accordance with ACR practice parameters or the EANM procedure guidelines for tumor imaging (version 3.0) [48]. Moreover, adherence to harmonization programs such as EARL (EANM Research Ltd) is strongly encouraged to ensure cross-scanner quantitative consistency [72]. Finally, the Lugano classification and Barrington et al. specifically advise that standard reconstructions should be used for semi-quantitative assessment, as advanced reconstructions (e.g., point-spread-function modeling, Bayesian penalized likelihood) can increase SUVmax values and alter the number of positive reads [38, 63].

### Reference region measurement and quantitative thresholds for meaningful change

The SUVmax of a reference organ (e.g., liver, mediastinal blood pool) not involved by disease should be measured on each scan to serve as an internal control, helping ensure that observed SUV changes in tumors reflect true biologic change rather than technical drift. Moreover, with careful protocol adherence, tumor SUVmax of a lesion has a within-subject coefficient of variation of approximately 10%. In a response assessment setting, SUV reductions > 25% and increases > 33% are unlikely to be due to measurement variability alone [73]. This intrinsic random variation of the SUVmax have to be consider for example when DS 4 and 5 are defined on semiquantitative data.

## Conclusions and Recommendations (Table 1; Fig. 2)

**Table 1:** FDG PET/CT Findings and CAR-T–Oriented Clinical Implications in DLBCL

Clinical time point	PET/CT assessment	Key PET metrics to report	Interpretation	CAR-T–oriented implication
Baseline at diagnosis	Initial staging PET/CT	Ann Arbor stage; extranodal sites; bulky disease; SUVmax; MTV, TLG (and Dmax if available)	Defines disease extent and metabolic burden	Identifies high-risk patients requiring closer monitoring and early multidisciplinary discussion
Interim PET/CT	Early response assessment	Deauville score; SUVmax of hottest lesion; (ΔSUVmax; liver SUVmax can be useful)	DS 1–3: CMR;DS 4: positive to be confirmed;DS 5: high-risk disease and potential treatment failure	DS 5 or inadequate ΔSUVmax should prompt early multidisciplinary review and consideration of CAR-T referral pathways
Suspected progression during therapy	Early PET if clinically indicated	New lesions and/or increase SUVmax of baseline lesions	Differentiate progression vs inflammation/infection	Biopsy recommended before treatment change; early CAR-T evaluation if refractory disease is confirmed
End-of-treatment PET/CT	Standard response assessment	Deauville score; Lugano category; SUVmax; lesion site; comparison with prior PET	DS 1–3: CMR;DS 4: positive to be confirmedDS 5: high probability of active disease	DS 5 should prompt CAR-T eligibility assessment (and biopsy if required)DS 4 requires confirmation (biopsy) before escalation
Relapse / refractory disease	Restaging PET/CT	Disease distribution; extranodal sites; SUVmax; MTV/TMTV; TLG; biopsy target	Defines tumor burden and guides biopsy	Supports CAR-T eligibility assessment and bridging strategy planning

*CMR* complete metabolic response, *Dmax* maximum tumor dissemination, *DS* Deauville score, *MTV* metabolic tumor volume, *SUVmax* maximum standardized uptake value, *TLG* total lesion glycolysis, *TMTV* total metabolic tumor volume

**Fig. 2:**
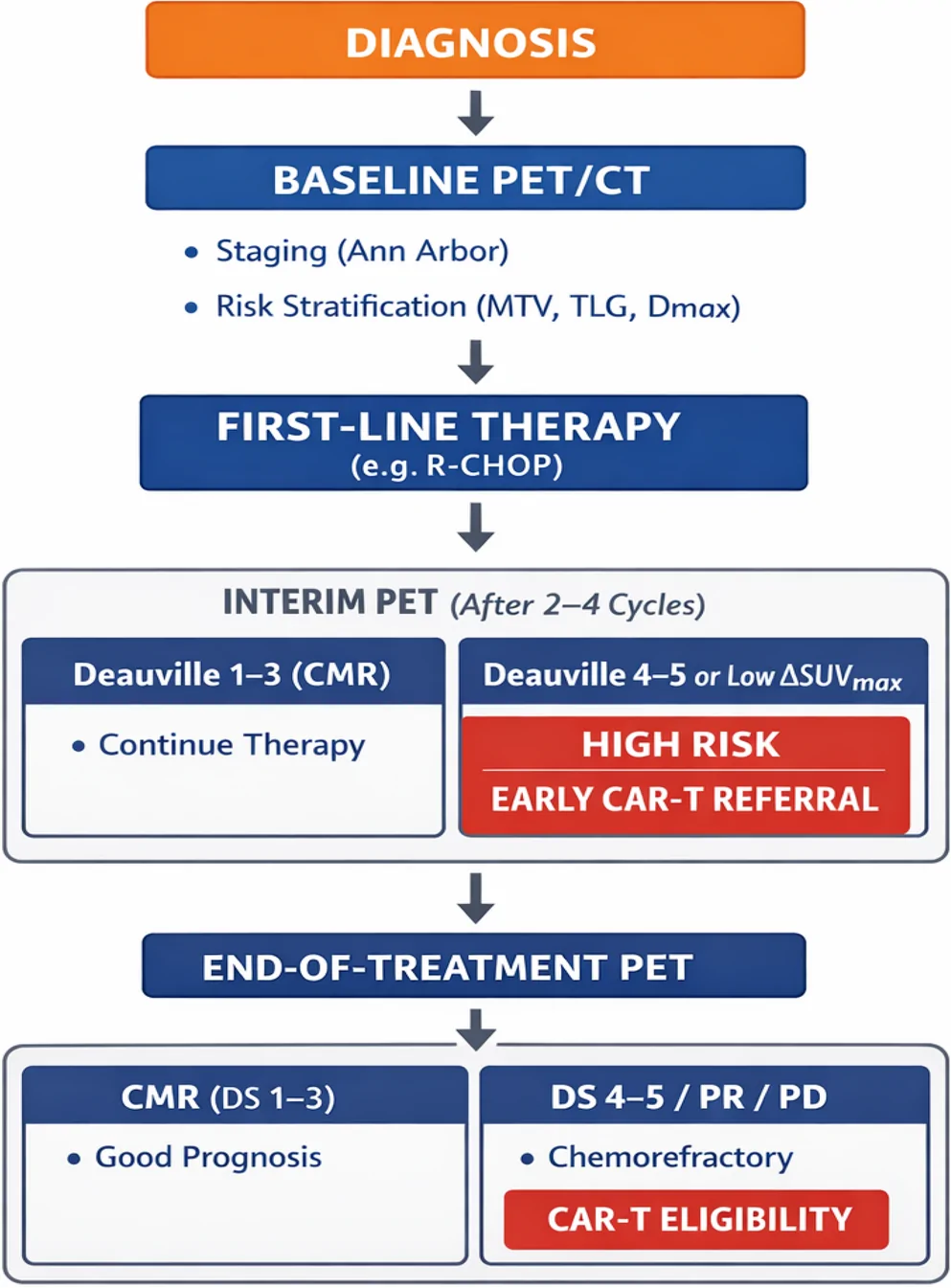
Early PET assessment for identification of CAR-T candidates in diffuse large B-Cell Lymphoma

On the basis of the available evidence and expert consensus, the following recommendations are proposed to optimize the use of [¹⁸F]FDG PET/CT in the management of DLBCL patients, particularly in the context of early identification and referral for CAR T-cell therapy.

1. **Early integration of PET/CT into risk-adapted clinical pathways**PET/CT should be considered a central tool not only for staging and response assessment but also for early risk stratification. Nuclear medicine physicians and hematologists should integrate PET-derived information from diagnosis onward to identify patients at high risk of relapsed/refractory disease and to facilitate timely referral for advanced therapies, including CAR T-cell therapy.
2. **Standardization of baseline PET/CT acquisition and reporting**Baseline PET/CT must be systematically performed for staging and should include quantitative parameters such as MTV, TLG, and, where feasible, Dmax. Among these, MTV is currently strongly recommended for routine reporting due to its robust and independent prognostic value. Harmonization of segmentation methods and broader availability of validated software tools are encouraged to enable consistent clinical implementation and the inclusion of the parameter in clinical report in baseline PET/CT.
3. **Minimization of time interval between baseline PET and treatment initiation**Given the aggressive biology of DLBCL, the interval between baseline PET/CT and treatment initiation should be minimized (within 1 month). If significant clinical progression is suspected, repeat imaging should be considered to ensure accurate disease assessment and avoid misinterpretation of treatment response.
4. **Systematic use of interim PET/CT for early response assessment**Interim PET/CT should be performed in all patients, preferably after 3–4 cycles of therapy (after 2 cycles in selected cases). DS remains the standard for visual assessment; however, it should be complemented by semiquantitative parameters, particularly ΔSUVmax, to improve risk stratification and reduce false-positive interpretations.
5. **Clear distinction between Deauville score 4 and 5**DS 5 should be regarded as the most reliable indicator of high-risk disease and potential treatment failure, warranting early consideration of second-line strategies, including CAR T-cell therapy. In contrast, DS 4 should be interpreted with caution due to its higher false-positive rate, and treatment escalation based solely on DS 4 should generally be avoided without further clinical confirmation.
6. **Mandatory inclusion of key quantitative metrics in PET response assessment reports**Response assessment PET/CT reports should consistently include the DS, SUVmax of the most metabolically active lesion, its anatomical location, the baseline highest SUVmax, the derived ΔSUVmax and the category of response according Lugano classification. When possible, liver SUVmax measurements should also be reported as an internal reference to improve interpretation reliability. These elements are essential for clinicians to appropriately stratify patients and guide therapeutic decisions.
7. **Biopsy confirmation before treatment modification**In cases where PET findings may lead to a change in therapeutic strategy, particularly in non-complete metabolic response, histological confirmation is strongly recommended to avoid overtreatment due to false-positive imaging findings.
8. **Central role of EOT PET/CT in guiding further management**EOT PET/CT, interpreted according to the DS and Lugano classification, remains the standard for response assessment. Patients with DS 1–3 should be considered in complete metabolic response, while those with DS 4–5 require individualized management, including biopsy confirmation and early evaluation for second-line therapies such as CAR T-cell therapy.
9. **PET/CT-driven identification of candidates for CAR T-cell therapy**Patients with primary refractory disease, early relapse, or inadequate metabolic response (particularly DS 5 and/or insufficient ΔSUVmax reduction) should be promptly evaluated for CAR T-cell therapy. Early activation of referral pathways is critical to maximize clinical benefit.
10. **Optimization of PET/CT methodology to ensure reproducibility**Strict adherence to standardized acquisition protocols is essential when using quantitative PET parameters. Key factors include consistent uptake time, use of the same scanner and reconstruction algorithms, and compliance with international harmonization standards (e.g., EANM/EARL). Reference organ measurements (e.g., liver SUV) should be routinely included to control for technical variability.
11. **Need for harmonized**,** consensus-driven clinical implementation**There is a clear need for standardized, widely adopted guidelines defining the optimal timing of PET/CT, required quantitative parameters, and their integration into CAR T-cell decision-making pathways. Multidisciplinary collaboration between nuclear medicine physicians and hematologists is essential to ensure consistent and effective clinical application.
12. **Future directions and research priorities**Further prospective studies are needed to validate PET-derived quantitative thresholds, clarify the prognostic role of dynamic changes over time, and define how these parameters can be incorporated into clinical decision algorithms. In particular, the role of PET in guiding early CAR T-cell referral and treatment sequencing warrants dedicated investigation.

## Conclusion

In conclusion, the current body of evidence are pushing PET/CT to evolve from a purely diagnostic tool to a fully integrated biomarker within personalized treatment strategies for DLBCL, enabling earlier identification of high-risk patients and improving access to potentially curative therapies such as CAR T-cell therapy. In this scenario, integration of clinicians and nuclear medicine physician competences is crucial to make available the best diagnostic and therapeutic path for the DLBCL patients.

## Data Availability

Not applicable.
